# Histological Analysis of the Prostate During the Human Fetal Period (12 to 22 Weeks Post-Conception)

**DOI:** 10.1590/S1677-5538.IBJU.2025.9915

**Published:** 2025-07-25

**Authors:** Edilaine Farias Alves, Carla Braga Mano Gallo, Waldemar Silva Costa, Luciano Alves Favorito, Francisco J. B. Sampaio

**Affiliations:** 1 Universidade do Estado do Rio de Janeiro Unidade de Pesquisa Urogenital Rio de Janeiro RJ Brasil Unidade de Pesquisa Urogenital - Universidade do Estado do Rio de Janeiro - Uerj, Rio de Janeiro, RJ, Brasil

**Keywords:** Prostate, Fetus, Growth and Development

## Abstract

**Purpose:**

The aim of this study was to quantitatively analyze the stromal components of the prostate (collagen, muscle fibers and elastic system fibers) during prostate development in normal human fetuses, in order to establish normal growth patterns.

**Materials and Methods:**

Twenty-one normal human fetuses, aged between 12- and 22-weeks post-conception (WPC), were studied. The fetuses were divided into three groups: G1, n = 7, 12 to 16 WPC; G2, n = 6, 17 to 19 WPC, and G3, n = 8, 20 to 22 WPC. The samples were fixed in 4% buffered formalin and processed for paraffin embedding for histochemical analysis. Five 5-micrometer-thick sections were taken from each sample and stained using histochemical techniques to identify the prostate stromal components. Histomorphometric analyses were carried out by evaluating the area density of the parameters analyzed, in percentage, using ImageJ software.

**Results:**

The quantitative analysis of collagen fibers (p = 0.0616) and elastic system fibers (p = 0.6049) showed no significant difference between groups. Smooth muscle fibers were higher in G2 (+82.38%) and G3 (+108.81%) when compared to G1, p = 0.0020. Linear regression of the entire fetal period studied (12 to 22 WPC) showed a proportional increase in muscle fibers in relation to gestational age (r2 = 0.3398; p = 0.0056). Linear regression revealed a strong negative correlation between collagen fibers and smooth muscle fibers in G3 (20 to 22 WPC) (r2 = 0.8209; p = 0.0019).

**Conclusions:**

Changes in the stromal components of the prostate are more significant from the end of the second trimester of pregnancy. Collagen fibers and elastic system fibers showed stable growth throughout the fetal period studied. The density of smooth muscle fibers increased about twofold with increasing fetal age.

## INTRODUCTION

The prostate is a fibromuscular and glandular organ, made up of approximately 70% glandular components, arranged in acini and ductules, and 30% stromal components, made up of fibromuscular tissue and a protein matrix (1, 2).

The prostate develops during the tenth week of gestation in response to the stimulus of androgenic hormones, mainly dihydrotestosterone, with complex and subsequent epithelial-mesenchymal interactions (3, 4). These hormones play an important role in prostate development and are potential stimulators of the differentiation and growth of the prostatic epithelium and stroma, being important in the structure and function of the prostate (3, 5, 6).

The human prostate undergoes numerous morphological changes from fetal development to old age (7). Prostate development takes place through several stages, ranging from the emergence of epithelial buds from the embryonic urogenital sinus to the differentiation of luminal and basal epithelial cells and secretory cytodifferentiation at the end of the second and third gestational trimesters, and is completed at puberty, in response to an increase in androgen production (8).

Previous studies have evaluated prostate growth in human fetuses, with and without congenital anomalies (9-14). However, studies evaluating the development of the stromal components of the fetal prostate are rare. Specific data on prostate growth during the human fetal period is relevant and of great importance to the urological community, as it can provide data on the biology of prostate development. Therefore, the aim of this study was to characterize, using quantitative methods, the growth and modifications of the stromal components of the prostate in normal human fetuses, from 12 to 22 weeks post-conception (WPC), in order to establish normal growth patterns.

## MATERIALS AND METHODS

The study was carried out in accordance with the Research Ethics Committee of the State University of Rio de Janeiro on experimentation on human subjects (IRB: 6.997.014, CAAE: 17321519.0.0000.5259).

We studied twenty-one normal human fetuses aged between 12 and 22 WPC, divided into three groups: G1 (n=7; 12 to 16 WPC); G2 (n=6; 17 to 19 WPC) and G3 (n=8; 20 to 22 WPC), from the collection of the Urogenital Research Unit of the State University of Rio de Janeiro. The fetuses were macroscopically well preserved, and the deaths were not related to the urogenital tract.

The gestational age of the fetuses was determined in WPC, according to the criterion of the largest foot length, which is the most acceptable and reliable parameter for estimating gestational age (15-18).

### Material and histological procedures

After gestational age determination, the fetuses were dissected using a stereoscopic lens with a magnification between 16 and 25x, and the urogenital block, containing the ureters, bladder, prostate and penis, was removed.

After collecting the samples, the prostate was separated from the other structures, fixed in 4% buffered formalin and routinely processed for paraffin embedding. After embedding, 5 µm-thick histological sections were made at 200 µm intervals to analyze the stromal components of the prostate. The sections were stained using the following histochemical methods: Hematoxylin-Eosin to assess tissue integrity, Picrosirius red to analyze collagen fibers, Masson's trichrome to analyze smooth muscle fibers, and Weigert's Resorcin-Fuchsin with prior oxidation to analyze the fibers of the elastic system.

### Histomorphometric analyses

For the histomorphometric analyses of the prostate stromal components we used the Image-J software, version 1.50i (http://www.imagej.nih.gov/ij). The analyses were carried out using photomicrographs taken with a microscope (BX51, Olympus America, Inc.) equipped with a digital camera (DP71, Olympus America, Inc., Melville, New York). For each analysis, the photomicrographs were captured and saved at a resolution of 2040 pixels x 1536 pixels, in TIFF format.

Quantitative analyses of the stromal components of the prostate were determined by the area densities of each component at the following magnifications: collagen fibers (400x), smooth muscle fibers (400x) and elastic system fibers (600x). For these analyses, five sections of each prostate were used and five random fields of each section were observed, totaling 25 fields in each prostate. The results for each field were obtained using the point-counting method, in which a grid of 100 points was superimposed on the photomicrographs using the "grid" tool, and the average quantification value was determined for the five sections studied from each prostate, with the results expressed as a percentage.

### Statistical Analysis

The Kolmogorov-Smirnov test was used to verify the normal distribution curve. The data was evaluated by analysis of variance (one-way ANOVA) with Tukey's post-test and the results were expressed as mean ± standard deviation. The mean values of each fetus were used for statistical analysis by linear regression, in which we evaluated the correlation coefficient (r) of WPC and the percentage of tissue area density of the prostatic stromal components. A p-value < 0.05 was considered statistically significant. All analyses were carried out using GraphPad Prism 5 software (GraphPad Software, San Diego, CA).

## RESULTS

**Figure 1:**
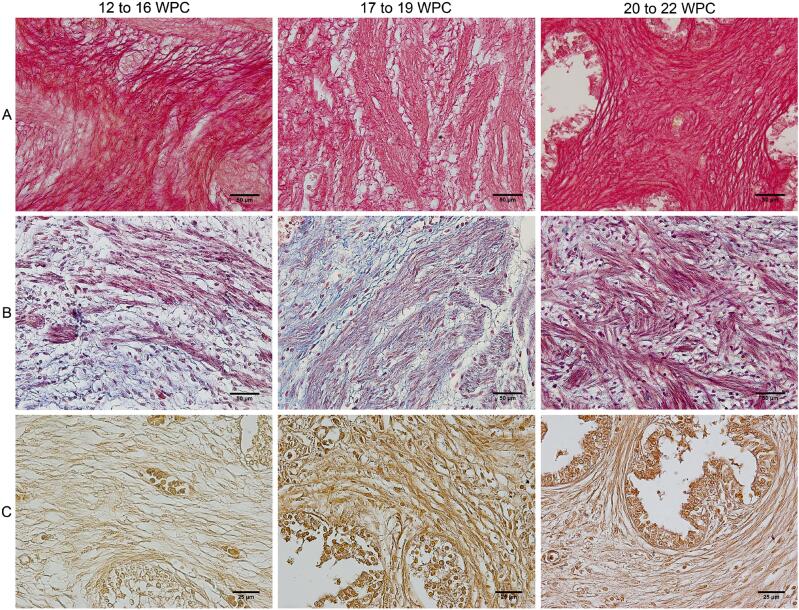
Photomicrograph of the prostate components in normal human fetuses.

The prostate of fetuses with 12 WPC already presented the stromal components differentiated (Figure-1).

Group 1 (12 to 16 WPC), Group 2 (17 to 19 WPC) and Group 3 (20 to 22 WPC). A) Collagen fibers, Picrosirius red, 400x. B) Smooth muscle fibers, Masson's trichrome, 400x. C) Elastic system fibers, Weigert's resorcin-fuchsin, 600x.

The results of the quantitative analyses in the three groups studied, G1 (12 to 16 WPC); G2 (17 to 19 WPC) and G3 (20 to 22 WPC), with mean and standard deviation, and the minimum and maximum values, for each group, are shown in Table-1.

**Table 1:** The table shows the analyzed parameters in the prostate of normal human fetuses according to fetal age in weeks post-conception (WPC).

Parameter	Group 1 (12 to 16WPC)	Group 2 (17 to 19WPC)	Group 3 (20 to 22WPC)	*p value*
Collagen Fibers	32.9 to 61.8% (mean = 46/SD+- 8.2)	22.4 to 49.9% (mean= 33.8 /SD+-9.7)	17.9 to 50.5% (mean=35.1 /SD+-9.1)	p=0.0616
Smooth Muscle Fibers	8.5 to 33.8% (mean =19.3 /SD+-9.7)	17.3 to 43.7% (mean=35.2/SD+- 9.2)	26.4 to 54.3% (mean=40.3/SD+-8.5)	p=0.0020
Elastic System Fibers	18.5 to 23.7% (mean =22.1 /SD+-2)	15.7 to 25.7% (mean=20.1 /SD+-3.6)	16 to 25.2% (mean=21.7 /SD+-3.5)	p=0.6049

Group 1 (n = 7; 12 to 16 WPC); Group 2 (n = 6; 17 to 19 WPC) and Group 3 (n = 8; 20 to 22 WPC)

SD = standard deviation.

**Figure 2:**
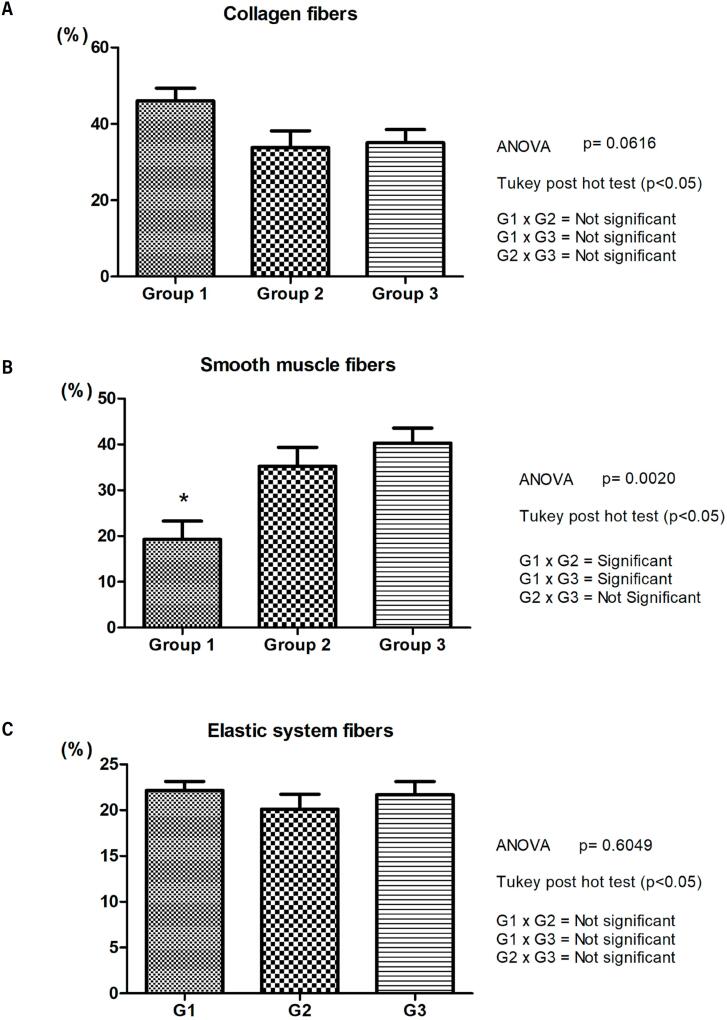
Graphs comparing the percentage of collagen fibers, smooth muscle fibers and elastic system fibers according to weeks post-conception (WPC).

A comparison of the growth of the stromal components of the prostate (collagen fibers, smooth muscle fibers and elastic system fibers) in the groups studied, showed a significant difference only in the muscular component of G1 when compared to the other groups (G1: 19.3 ± 9.74%; G2: 35.2 ± 9.21%; G3: 40.3 ± 8.54%; p=0.0020). Collagen fibers (G1: 46.0 ± 8.24%; G2: 33.8 ± 9.67%; G3: 35.1 ± 9.11%; p=0.0616) and elastic system fibers (G1: 22.1 ± 1.98%; G2: 20.1 ± 3.63%; G3: 21.7 ± 3.53%; p=0.6049) showed no significant difference between the groups (Figure-2).

Group 1 (12 to 16 WPC), Group 2 (17 to 19 WPC) and Group 3 (20 to 22 WPC). Data were expressed as the means ± standard deviation and were compared by one-way ANOVA and Tukey post hoc test, P<0.05. The symbol [*] indicates significant difference for G2 and G3.

**Figure 3:**
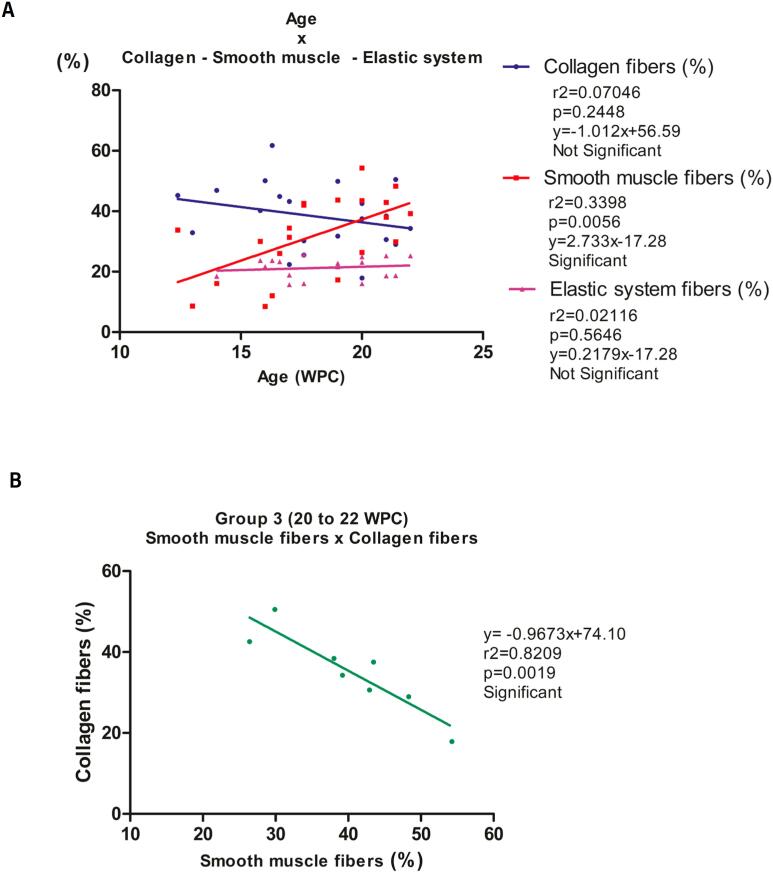
Correlation between collagen fibers, smooth muscle fibers and elastic system fibers, in percentage, with fetal age, during the fetal period studied (12 to 22 weeks post-conception – WPC).

The linear regression analysis showed a significant positive correlation between muscle and gestational age throughout the fetal period studied (r2 = 0.3398; p = 0.0056). Collagen (r2 = 0.07046; p = 0.2448) and elastic system fibers (r2 = 0.02116; p = 0.5646) showed no significant correlation. Linear regression analysis also revealed a strong significant negative correlation in G3 between muscle fibers and collagen (r2 = 0.8209; p = 0.0019) (Figure-3).

The points plotted represent the mean values obtained for each week studied.

A) Correlation during entire fetal period studied. Linear regression indicated that the smooth muscle fibers are correlated significantly and positively with fetal age (r2 = 0.3398; p = 0.0056).

B) Correlation during second trimester (Group 3, 20 to 22 WPC). Linear regression indicated a strong negative correlation between collagen fibers and smooth muscle fibers from 20 to 22 WPC (r2 = 0.8209; p = 0.0019).

## DISCUSSION

The prostate is a fibromuscular and glandular organ of great clinical interest, since pathologies such as prostate cancer and benign prostatic hyperplasia are common in individuals over 40 years of age. Studies of prostate development in normal human fetuses during the second gestational trimester are rare and may be relevant due to the importance of this fetal period in the growth and development of the embryo.

As the changes during embryonic development are rapid and numerous, the fetuses were divided into three groups according to gestational age. Prostate development undergoes various differentiations, and a previous study observed intense prostate development between the 12th and 20th weeks (19).

Lunacek et al. (20) evaluated fetal prostates between 11 and 40 weeks of gestation and observed that the first signs of glandular tissue development appeared in the 11th week. A recent study evaluated the development of the urogenital tract in normal human fetuses and observed the appearance of the prostate at the 12th week with the appearance of prostatic buds (12). In the present study, we observed that the prostatic components analyzed here were already present from the 12th WPC onwards.

The prostatic stroma is made up mainly of collagen and smooth muscle.11 Between 6 and 13 weeks, the amount of connective tissue around the acini increases consistently (21).

Our study showed a tendency for the percentage of collagen fibers to decrease throughout the fetal period studied (12 to 22 WPC), however, with no significant difference between the groups (Figures-1A and 2A). As during this period there is an increase in the volume of the prostate (13, 14), we can assume that there was no increase in collagen fibers during this period. On the other hand, we found that muscle fibers showed a significant increase according to the groups studied. There was a greater quantity of smooth muscle fibers than collagen up to the 22nd WPC (Figure-3).

The main and most obvious component of the normal prostate stroma are the smooth muscle fibers found around the acini (22-24). Studies suggest that the prostatic epithelium induces smooth muscle differentiation in the mesenchyme, as ductal branching is more numerous where smooth muscle cells are abundant (8, 25).

A study using alpha-actin immunomarker observed an increased expression of smooth muscle in the fetal prostate (8). In our study, the percentage of smooth muscle fibers showed a significant difference between the groups studied, being higher in groups G2 and G3, when compared to G1, with a significant increase in the percentage of this parameter with increasing of gestational age (Figures-1B and 2B). The linear regression analysis revealed a significant positive correlation between age and smooth muscle fibers when analyzed throughout the fetal period studied (12 to 22 WPC), suggesting a proportional growth of this component in relation to gestational age (Figure-3A). We observed a significant negative correlation between smooth muscle and collagen fibers around the 20th WPC, indicating that as the smooth muscle increases, the collagen fibers decrease (Figure-3B). These findings show that muscle fiber density increases proportionally with age. Gallo et al. (26) observed in the fetal penis a proportional increase in muscle in relation to age, and a relationship between muscle and collagen from the 22nd WPC onwards. It is known that the extracellular matrix is made up of various components, including collagen and elastic system fibers, the latter of which are involved in the structure and organization of the prostatic tissue (27). A previous study evaluating the elastic system fibers in the penis, observed the presence of this component from the 13th WPC onwards and showed its proportional growth with gestational age (26, 28). Our study showed a stable percentage of elastic system fibers in the prostate in the different groups (Figures-1C, 2C and 3A).

## CONCLUSIONS

The changes observed in the stromal components of the prostate were more significant at the end of the second gestational trimester. The density of smooth muscle fibers increased about twofold with gestational age, indicating a proportional growth to the fetal age (12 to 22 WPC) with a strong positive significant correlation. A strong negative correlation was observed between collagen fibers and smooth muscle fibers from 20 to 22 WPC.
